# Effect of betaine supplementation on cycling sprint performance

**DOI:** 10.1186/1550-2783-9-12

**Published:** 2012-04-03

**Authors:** J Luke Pryor, Stuart AS Craig, Thomas Swensen

**Affiliations:** 1 Department of Kinesiology, University of Connecticut, Storrs, CT 06269, U-1110, USA; 2 DuPont Nutrition & Health, Tarrytown, NY, USA; 3 Ithaca College, Ithaca, NY, USA

**Keywords:** Anaerobic power, Ergogenic aid, Creatine, Wingate

## Abstract

**Purpose:**

To examine the effect of betaine supplementation on cycling sprint performance.

**Methods:**

Sixteen recreationally active subjects (7 females and 9 males) completed three sprint tests, each consisting of four 12 sec efforts against a resistance equal to 5.5% of body weight; efforts were separated by 2.5 min of cycling at zero resistance. Test one established baseline; test two and three were preceded by seven days of daily consumption of 591 ml of a carbohydrate-electrolyte beverage as a placebo or a carbohydrate-electrolyte beverage containing 0.42% betaine (approximately 2.5 grams of betaine a day); half the beverage was consumed in the morning and the other half in the afternoon. We used a double blind random order cross-over design; there was a 3 wk washout between trials two and three. Average and maximum peak and mean power were analyzed with one-way repeated measures ANOVA and, where indicated, a Student Newman-Keuls.

**Results:**

Compared to baseline, betaine ingestion increased average peak power (6.4%; p < 0.001), maximum peak power (5.7%; p < 0.001), average mean power (5.4%; p = 0.004), and maximum mean power (4.4%; p = 0.004) for all subjects combined. Compared to placebo, betaine ingestion significantly increased average peak power (3.4%; p = 0.026), maximum peak power max (3.8%; p = 0.007), average mean power (3.3%; p = 0.034), and maximum mean power (3.5%; p = 0.011) for all subjects combined. There were no differences between the placebo and baseline trials.

**Conclusions:**

One week of betaine ingestion improved cycling sprint power in recreationally active males and females.

## Background

Betaine is a nutrient found in a variety of animals, plants, and microorganisms [1]. It is a component of many foods, with whole grains (e.g., wheat, rye), spinach, shellfish and beets [2] being rich sources. As an organic osmolyte, betaine or trimethyl glycine, protects cells under stress, such as dehydration; it is also a source of methyl groups, via the methionine cycle, in many key biochemical pathways [1]. Betaine, therefore, plays an important role in several aspects of human health and nutrition and studies show that diets high in betaine decrease disease risk [1,3-5].

In addition to improving health, betaine may also improve sport performance. Since betaine is an osmolyte that protects cells under stress [6,7], initial studies on the potential ergogenicity focused on the acute effects of betaine ingestion on performance in the heat [8,9]. In one study, subjects ran in a heated environment (31.1°C) for 75 minutes at 65% of VO_2_max followed by a performance run at 84% of VO_2_max to volitional exhaustion [8]. Time to exhaustion was 16 to 21% (32 to 38 sec) greater when beverages with betaine or betaine and carbohydrate were consumed, respectively, but the changes were statistically insignificant (p ≥ 0.12). In the other study, subjects completed a 15 min cycling time trial after riding for 2 hr at 60-75% VO_2_max in the heat [9]; immediately after the time trial, isometric leg strength was also examined. Acute consumption of either a carbohydrate or a betaine and carbohydrate beverage before the test improved time trial performance by 10 and 14%, respectively, relative to a water control trial; there was no difference between the carbohydrate and carbohydrate and betaine trials. Isometric leg strength, however, was significantly greater after the betaine trials compared to the non-betaine trials.

This latter result catalyzed a series of inquires on the chronic effects of betaine ingestion (2 weeks) on various indices of strength and power [10,11]. The assumption being that since betaine is a methyl donor [1], it could theoretically boost creatine stores in the musculature, and therefore, improve strength and power [10]. Chronic betaine ingestion (at least 2.5 g^.^d^-1 ^for 14 d) significantly improved bench press repetitions, volume load, throw power, isometric bench press force, vertical jump power, isometric squat force, and muscle endurance during a squat exercise [10-12]. Despite enhancing the aforementioned indices of lower extremity strength and power, chronic betaine ingestion did not improve Wingate anaerobic power [10]. The inability of betaine to enhance cycling sprint performance, as measured with the Wingate anaerobic power test, may be related to the duration of the test and the amount of recovery between trials. Perhaps the 30 sec Wingate test and the 5 min recovery period between trials were too long to fully assess betaine's putative ability to enhance sport specific strength and power, both of which contribute significantly to Wingate performance. A series of shorter work bouts interspersed with shorter periods of active recovery may be a more applicable test of betaine's potential to enhance anaerobic power while cycling. To that end, our purpose was to examine the effect of one week of betaine ingestion on anaerobic power as measured with a series of four, 12 sec work bouts on the cycle ergometer.

## Methods

### Subjects

Sixteen college-aged males (n = 9) and females (n = 7) volunteered to participate in this study; their mean ± SD for age, height, and weight were: 19 ± 0.8 y, 172 ± 12.0 cm, and 75 ± 14.9 kg and morphological data are present in Table 1. All subjects were free of lower body musculoskeletal injury and reported no limitations to exercise. Subjects were informed of the experimental procedures and known risks, and signed an informed consent approved by the Ithaca College Human Subjects Review Board prior to participation.

**Table 1:** Body Composition

Variable	Baseline	Placebo	Betaine
Body Weight (kg)	75.1 ± 14.9	74.9 ± 14.9	75.4 ± 14.9
Free Fat Mass (kg)	60.1 ± 14.6	59.8 ± 14.6	59.7 ± 14.5
Fat Mass (kg)	15.0 ± 0.3	15.1 ± 0.3	15.7 ± 0.4
Percent Fat Mass	20.1 ± 10.5	20.2 ± 10.4	20.9 ± 10.9
Total Body Water (kg)	44.0 ± 10.7	43.8 ± 10.7	43.7 ± 10.6

Data are mean ± SD

* p < 0.05 compared to corresponding baseline value

^# ^p < 0.05 compared to corresponding placebo value

### Experimental design

This investigation examined the effects of two drink solutions on cycling sprint performance with a double blind cross-over design. The placebo was a commercial carbohydrate-electrolyte beverage (Wegmans MVP), whereas the same carbohydrate-electrolyte beverage with 2.5 g of betaine (minimum purity is 99%; BetaPower™ DuPont Nutrition & Health, Tarrytown, NY) was the experimental drink. Since betaine is colorless and tasteless, subjects could not differentiate between the two solutions. Furthermore, to ensure drink anonymity, all cap ties were broken prior to consumption. Subjects completed three cycling sprint tests, the first of which served as a baseline measure. Subjects were match-paired based upon maximum peak power and assigned to consume either the placebo or betaine beverage. They were instructed to consume approximately half (295 mL) of their respective beverage twice a day for seven days, after which they were tested again. The last drink was consumed the morning of the test day and all testing sessions took place in the evening. Following a three-week washout phase, a second seven day supplementation period with the opposite beverage occurred followed by the third testing session. Prior to every laboratory session, we used the Tanita 350 bioimedance body fat analyzer to assess the subjects' weight, total body water, fat free mass, and percent body fat (BF 350; Tanita Corporation of America, Inc. Arlington Heights, IL). This unit is valid and reliable [13-16].

### Performance testing

Prior to every sprint test, subjects pedaled at a self-selected pace against a light resistance for 5 min to warm up with two to three interspersed sprints of short duration. The sprint test followed which consisted of four, 12 sec work bouts on a Monark 834 E ergometer (Varberg, Sweden) against a resistance equal to 5.5% of body weight. Each work bout was separated by 2.5 min of cycling at zero resistance. At the completion of the test, subjects continued to pedal at zero resistance for 2.5 min to cool down. The ergometer was equipped with toe clips, seat height was standardized for each subject to allow for 10-15° of knee flexion, and vigorous verbal encouragement was provided for all tests. SMI Power software (Sports Medicine Industries, St. Cloud, MN) interfaced with the ergometer with an OptoSensor 2000 infrared sensor (Sports Medicine Industries, St. Cloud, MN) collected data every second. The sensor was calibrated before every testing session.

The following variables were measured during each sprint test: average peak power, maximum peak power, average mean power, and maximum mean power. Average peak and average mean power were calculated across the four work bouts in each sprint test; maximum peak and mean power were the highest values for the respective variables in any sprint test. Peak power was calculated as the highest power output over any five-second interval during a sprint test. The coefficient of variation for average peak power, maximum peak power, average mean power, and maximum mean power across two tests completed on separate days was assessed in a series of pilot studies (n = 6) and were 1.3, 1.8, 1.3, and 1.6%, respectively.

### Statistical analyses

Data were analyzed using one-way repeated measures ANOVA. Where indicated, a Student Newman-Kuels test was used to identify specific differences (SigmaPlot v11, Systat Software Inc, San Jose, CA); alpha was set at 0.05 for all tests. Data are presented as mean ± SD.

## Results

**Figure 1:**
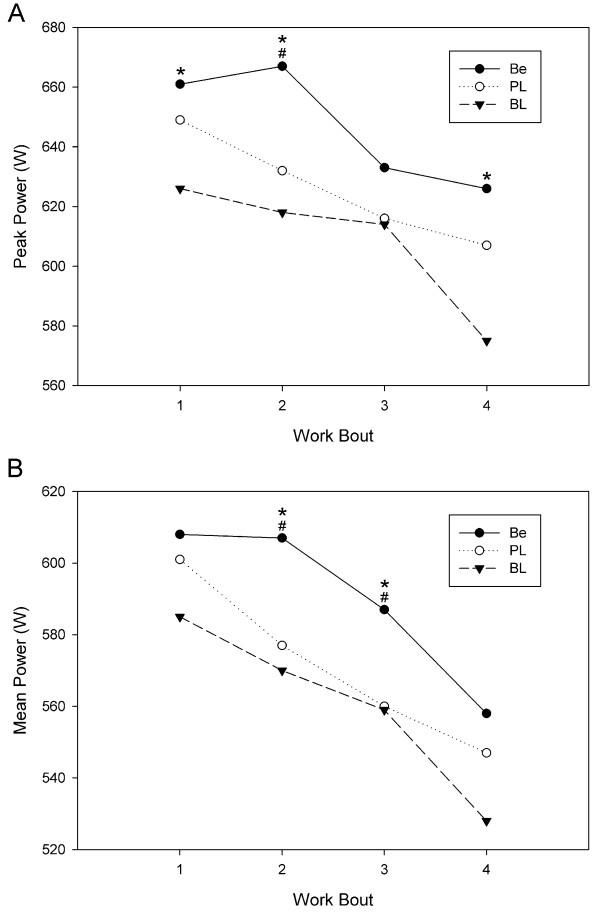
**Individual cycle runs power comparison for all subjects**. A: peak power; B: mean power. * p < 0.05 compared to corresponding baseline value. ^# ^p < 0.05 compared to corresponding placebo value. W = watts, BL = baseline, PL = placebo, Be = betaine.

**Figure 2:**
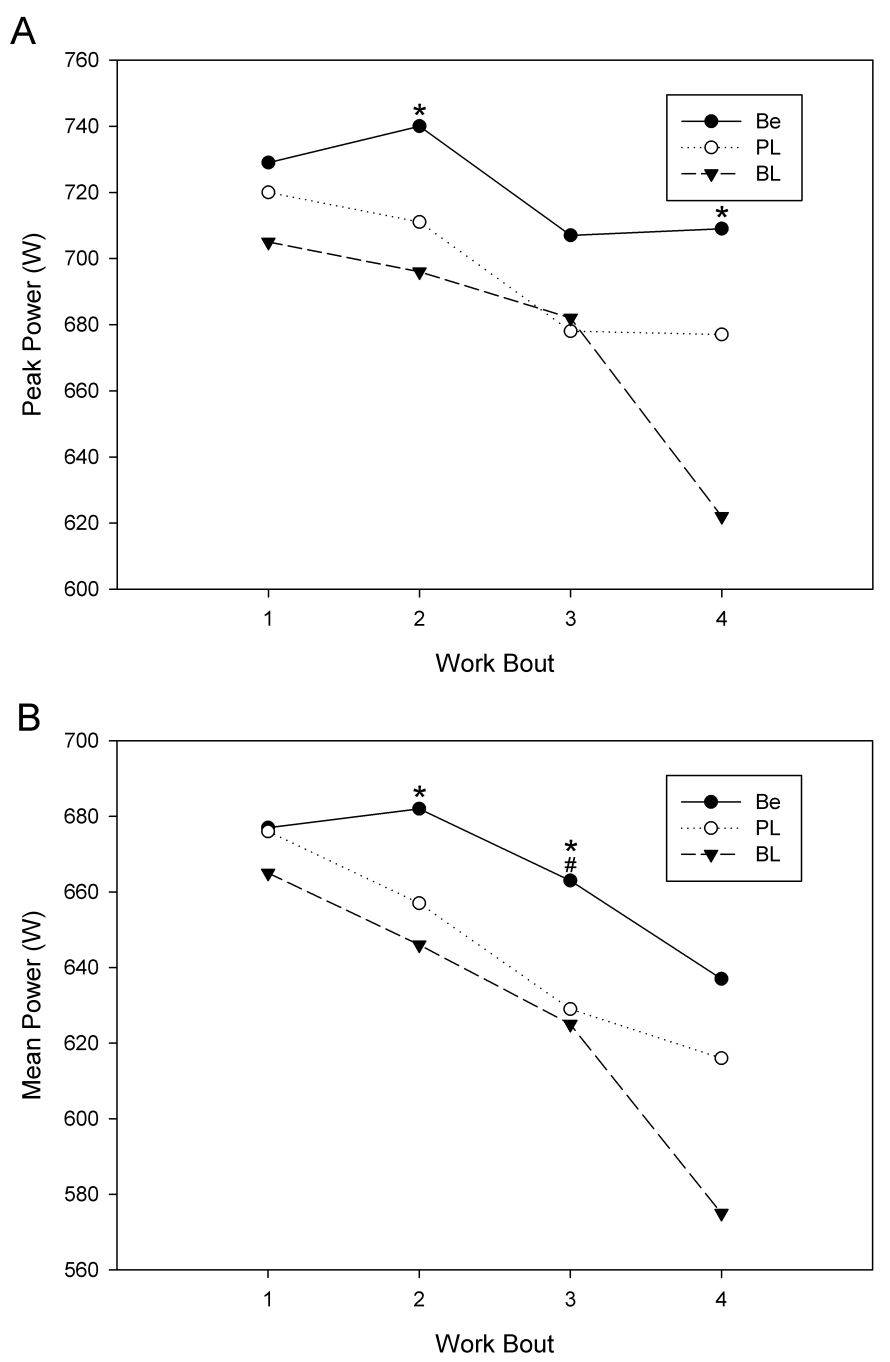
**Individual cycle runs power comparison for males**. A: peak power; B: mean power. * p < 0.05 compared to corresponding baseline value. ^# ^p < 0.05 compared to corresponding placebo value. W = watts, BL = baseline, PL = placebo, Be = betaine.

**Figure 3:**
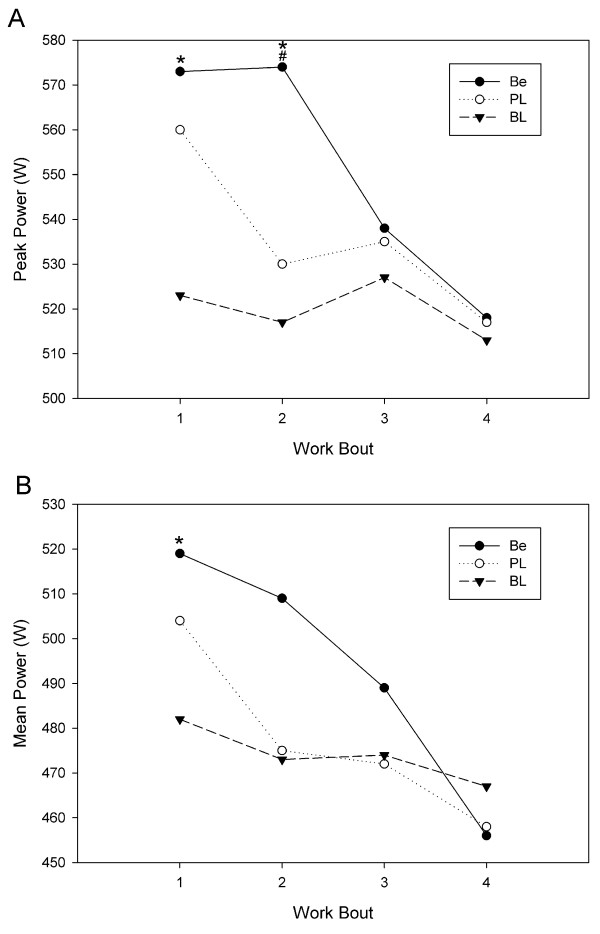
**Individual cycle runs power comparison for females**. A: peak power; B: mean power. * p < 0.05 compared to corresponding baseline value. ^# ^p < 0.05 compared to corresponding placebo value. W = watts, BL = baseline, PL = placebo, Be = betaine.

Based on the mean and SD for maximum peak power from the pilot study and an a priori assumption that a 4% change in power pre- to post-supplementation is meaningful, we used GPOWER software (Bonn, FRG) to determine that a sample size of 14 was needed to give us a power of 0.80 with an alpha of 0.05. Table 2 shows the mean and SD for average peak power, maximum peak power, average mean power and maximum mean power. Figures 1, 2, and 3 demonstrate mean and peak power across trials and gender. Compared to baseline, betaine ingestion increased average peak power (6.4%; p < 0.001), maximum peak power (5.7%; p < 0.001), average mean power (5.4%; p = 0.004), and maximum mean power (4.4%; p = 0.004) for all subjects combined. Compared to placebo, betaine ingestion significantly increased average peak power (3.4%; p = 0.026), maximum peak power (3.8%; p = 0.007), average mean power (3.3%; p = 0.034), and maximum mean power (3.5%; p = 0.011) for all subjects combined. There were no differences between the placebo and baseline trials. There were no differences across time or between conditions for any of the body composition variables.

**Table 2:** Combined power (watts) comparison for all subjects

Variable	Baseline	Placebo	Betaine
Peak Power			
Average	608 ± 140	626 ± 133	647 ± 144*^#^
Maximum	644 ± 144	656 ± 141	681 ± 145*^#^
Mean Power			
Average	560 ± 133	571 ± 126	590 ± 138*^#^
Maximum	596 ± 138	601 ± 131	622 ± 141*^#^

Data are mean ± SD

* p < 0.05 compared to corresponding baseline value

^# ^p < 0.05 compared to corresponding placebo value

## Discussion

Our purpose was to examine the effect of one week of betaine ingestion on anaerobic power as measured with a series of four, 12 sec work bouts. We found that one week of betaine ingestion (2.5 g^.^d^-1^) improved sprint performance by 5.5 ± 0.8% compared to baseline and 3.5 ± 0.2% compared to the carbohydrate placebo. These results contrast with data from Hoffman et al. [10], who reported daily consumption of 2.5 grams of betaine mixed with a commercially available carbohydrate beverage for 15 days did not enhance peak power, mean power, rate of fatigue, or total work across two Wingate trials separated by 5 min of active rest. One likely explanation for some of the difference in the results between the studies is the nature of the sprint test. Our subjects completed more sprints (4 vs. 2) of a shorter duration (12 vs. 30 sec) that were interspersed with shorter periods of active recovery (2.5 vs. 5 min) relative to the subjects in Hoffman et al. [10]. Experimental design may also account for some of the difference between the studies. Hoffman et al. [10] used a randomized repeated measures design, whereas we used a cross-over repeated measures design. Cross-over designs reduce the effects of confounding covariates and are more efficient statistically.

The likely mechanisms behind the increased power output we measured are related to methylation and osmolyte effects. Betaine supplementation may have elevated intramuscular creatine stores, increased muscle growth, or protected the muscle cells from stress-induced damage. The creatine hypothesis is attractive and supported by studies on betaine metabolism. In short, the liver enzyme betaine homocysteine methyltransferase transfers a methyl group from betaine to homocysteine, thereby producing dimethylglycine and methionine. The latter is then converted to S-adenosylmethionine (SAM), which subsequently acts as a methyl donor during creatine synthesis [17]. Studies show that betaine ingestion increases serum methionine, while betaine injection increases red blood cell SAM concentrations [18,19]. Our observed changes in sprint performance, moreover, are consistent with the performance effects of creatine supplementation, as shown in a meta-analysis [20]. Across 100 studies, creatine supplementation improved performance parameters by 5.7 ± 0.5% compared to baseline, whereas corresponding placebo effects were 2.4 ± 0.4%. More specifically, the meta-analysis showed that creatine supplementation improved lower extremity power by 5.6 ± 0.6% relative to baseline, which is similar to the 5.5 ± 0.8% increase we measured.

It is unlikely, however, that the amount of betaine consumed by our subjects (2.5 g^.^d^-1 ^for 7 d) elicits the same effect as the typical daily dosage of creatine during the loading phase of approximately 25 grams. This conjecture is supported by recently published data showing that 2 g^.^d^-1 ^of betaine for 10 day did not increase phosphorylcreatine levels compared to 20 g^.^d^-1 ^of creatine for 10 day [21]. This study also showed that betaine supplementation did not increase squat and bench press 1 RM or bench and squat power, findings that are inconsistent with data from earlier studies [10-12]. Direct comparison among the studies is difficult. Betaine dosage was lower in the recent study (2 vs 2.5 g^.^d^-1^), supplementation time was shorter (10 vs 15 d) and power output was not assessed until 3-5 d after supplementation ended compared to immediately afterwards [10,11].

Last, betaine supplementation may have enhanced sprint performance by acting as an osmolyte to maintain cell hydration and function under stress more effectively than placebo. Organic osmolytes are accumulated in cells when tissues are subjected to stress [6,22]. They help cells maintain optimal osmotic pressure, and allow proteins to maintain native folded conformation and stability without perturbing other cellular processes. Betaine helps maintain cell homeostasis by preventing formation of stress granules and keeping the mRNA associated machineries going under chronic hypertonicity [23]. Betaine is one of the most effective osmolytes, shown to facilitate a protective monolayer of water around biopolymers, enhance muscle cell survival and protein synthesis, and maintain myosin ATPase activity during periods of stress [24-26]. It may be that any or all of the aforementioned roles of betaine contributed to the 5.5% increase in power we observed.

## Conclusion

We found that one week of betaine supplementation increased peak and mean anaerobic power by approximately 5.5% compared to baseline measures in recreationally active college age men and women. The magnitude of this change is similar to the change in anaerobic power following creatine supplementation. Future research should elucidate the mechanism of improved performance via betaine supplementation.

## Competing interests

JLP and TS declare that they have no competing interests and will not benefit from the results of the present study. SASC is an employee of DuPont Nutrition & Health. Publication of these findings should not be viewed as endorsement by the investigators, Ithaca College, the University of Connecticut, or the editorial board of the Journal of the International Society of Sport Nutrition.

## Authors' contributions

JLP participated in drafting, editing, and submitting the manuscript. SASC assisted with study design, statistical analysis and critically reviewed the manuscript for intellectual content. TS supervised the research group, ran the statistical analysis, interpreted data, and was involved with manuscript drafting. All authors read and approved the final manuscript.
